# Data describing lack of effects of 17α-ethinyl estradiol on mammary gland morphology in female mice exposed during pregnancy and lactation

**DOI:** 10.1016/j.dib.2017.07.062

**Published:** 2017-07-27

**Authors:** Charlotte D. LaPlante, Laura N. Vandenberg

**Affiliations:** Department of Environmental Health Sciences, School of Public Health and Health Sciences, University of Massachusetts, Amherst, USA

**Keywords:** Xenoestrogen, Mammary gland, Lactation, Morphometric, Endocrine disruptor, Maternal

## Abstract

Ethinyl estradiol (EE) is a synthetic estrogen used in pharmaceutical contraceptives. In many studies evaluating estrogenic endocrine disruptors, EE is used as a positive control for estrogenicity. However, the effects of EE often differ from the effects of other xenoestrogens, suggesting that these other compounds might act via distinct mechanisms. Reported here are data describing the effect of low doses of EE during pregnancy and lactation on the morphology of the lactating mammary gland in CD-1 mice. The data suggest that these low doses have few if any discernable effects on mammary gland morphology. Alterations to cell proliferation and the expression of estrogen receptor (ER)α were also not observed. These companion data were collected from the same females analyzed for effects of EE on maternal behavior and brain recently published in Reproductive Toxicology (Catanese & Vandenberg, 2017).

**Specifications Table**

**Table:** 

Subject area	*Biology*
More specific subject area	*Endocrinology, reproductive science, endocrine disruptors*
Type of data	Figures, graphs Whole mount mammary glands Histological stain: Hematoxylin & Eosin Immunohistochemistry: Ki67 (marker of proliferation) and Estrogen Receptor α qRT-PCR: Esr1
How data was acquired	Zeiss AxioImager dissection microscope (whole mount glands) Zeiss Axio Oberserver.Z1 inverted microscope (histology and immunohistochemistry) Zeiss high resolution color camera
Data format	*Primary data, quantified and analyzed graphs*
Experimental factors	*Exposure of female CD-1 mice to 0.01 or 1 μg ethinyl estradiol/kg/day from pregnancy day 9 through lactational day 20; oral route of exposure* *Mammary glands collected on lactational day 21* *Whole mount mammary glands stained with carmine alum; mammary glands fixed in neutral buffered formalin, paraffin embedded and sectioned*
Experimental features	*Assessment of mammary gland morphology; quantification of epithelial cell proliferation; expression of estrogen receptor α in females exposed to vehicle (control) compared to females exposed to one of two doses of ethinyl estradiol*
Data source location	*Amherst, MA, USA*
Data accessibility	*Data are present in this article*

**Value of the data**

• Many studies examining the effects of estrogenic endocrine disrupting chemicals use EE as a positive control for estrogenicity
• Although high doses of pharmaceutical estrogens are known to disrupt lactation in rodents and women, the effects of low doses are not well described
• These data, together with data published elsewhere, can be used to identify endpoints that are sensitive and insensitive to xenoestrogens in females exposed during pregnancy and lactation

## 1 Data

**Fig. 1:**
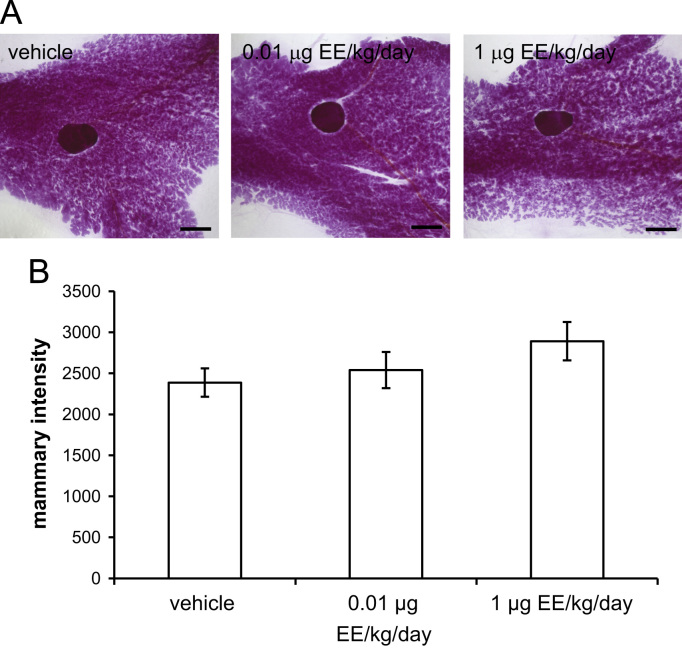
EE treatment does not affect mammary gland morphology at lactational day 21. **A)** Representative whole mount mammary glands collected from females exposed to vehicle, 0.01 or 1 μg EE/kg/day from pregnancy day 9 through lactational day 20. Mammary glands were collected on lactational day 21, prior to weaning, fixed and stained with carmine alum. Zeiss ZEN software was used to quantify intensity of gland (a measure of epithelial density) at four discrete locations. Scale bars represent 2 mm. **B)** Quantification of data collected from whole mounts. Intensity has arbitrary units. Statistical significance was evaluated using 1-way ANOVA and Bonferroni posthoc tests, and no significant differences between groups were revealed.

The mammary gland whole mounts and histological sections displayed in Fig. 1A are representative images from female CD-1 mice exposed to vehicle, 0.01 or 1 μg ethinyl estradiol (EE)/kg/day from pregnancy day 9 through lactational day 20. Quantification of mammary gland intensity, a measure of the epithelial density, reveals modest but non-significant decreases in the amount of mammary epithelium in EE-treated females (represented by higher intensity values) (Fig. 1B).

**Fig. 2:**
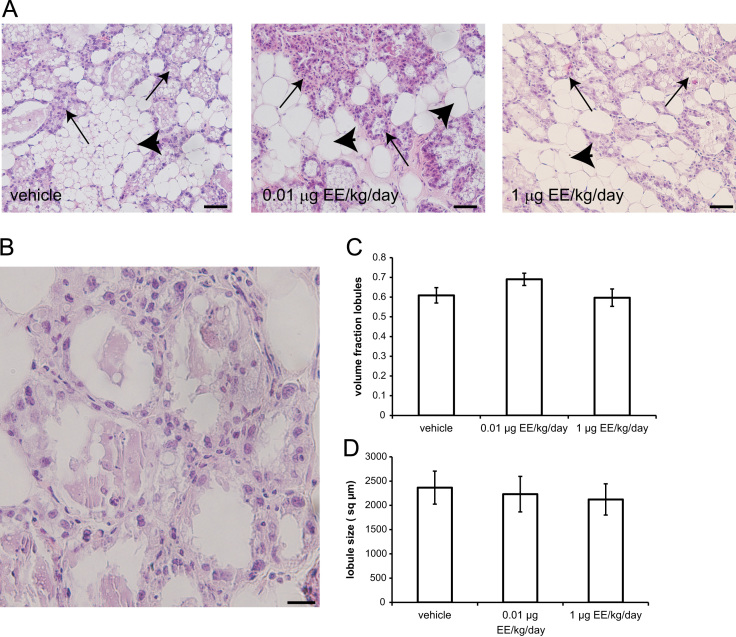
EE treatment does not affect histomorphological parameters on the lactating mammary gland. **A)** Representative histological sections stained with hematoxylin and eosin collected from females exposed to vehicle, 0.01 or 1 μg EE/kg/day from pregnancy day 9 through lactational day 20. Arrows indicate lobuloalveolar structures, arrowheads indicate adipose tissue. Scale bar represents 50 μm. **B)** A higher magnification image demonstrating lobules of varying size. Scale bar represents 20 μm. **C)** Quantification of data collected from histological sections evaluating the volume fraction of the mammary gland comprised of lobuloalveolar units. **D)** Quantification of lobule size. Neither the volume fraction of lobuloalveolar units nor the lobule size was affected by EE treatment.

To further investigate the effects of EE on morphology of the lactating mammary gland, we evaluated two histological characteristics in fixed tissue at lactational day 21: the volume fraction of the mammary gland comprised of lobuloalveolar structures and lobule size (Fig. 2A,B). There was no effect of EE treatment on either parameter (Fig. 2C,D).

**Fig. 3:**
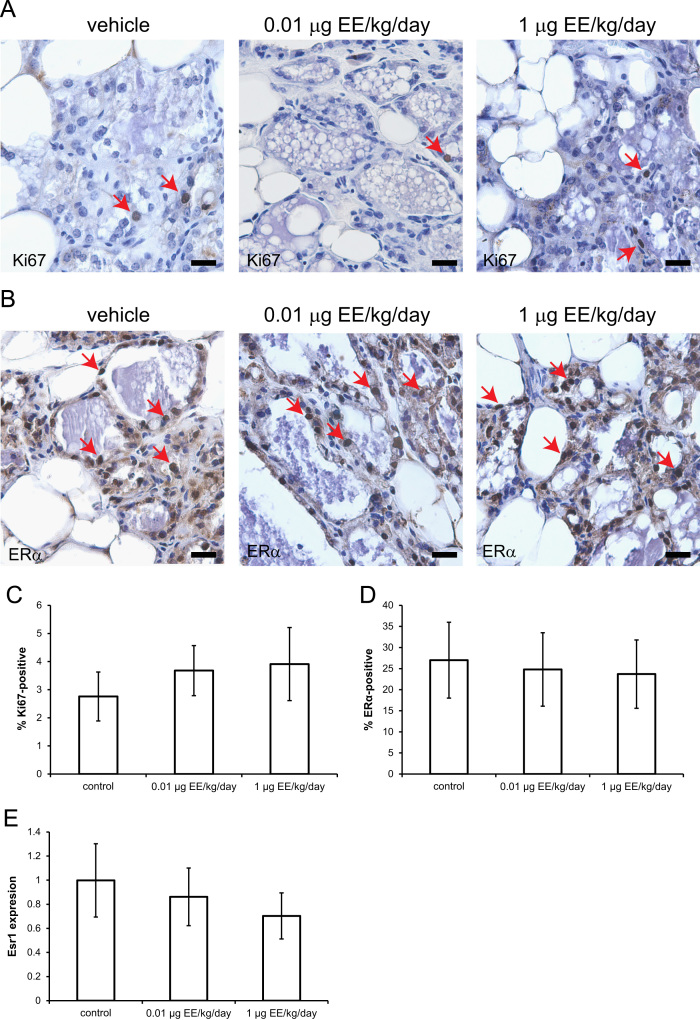
EE does not alter epithelial cell proliferation, expression of ERα, or expression of Esr1. Immunohistochemical evaluations of Ki67 **(A)** and ERα **(B)** were performed at LD21. Scale bar in both panels represents 20 μm, red arrows indicate positive cells. **C)** Quantification of Ki67 expression revealed no effect of EE treatment on epithelial cell proliferation. **D)** The number of ERα-positive mammary epithelial cells was also not altered by EE treatment. **E)** Esr1 expression, normalized to β-actin expression, was not affected by EE treatment.

Finally, we examined the effect of EE treatment on cell proliferation (evaluated by quantifying the number of cells expressing Ki67, Fig. 3A) and the number of cells positive for estrogen receptor (ER)α (Fig. 3B). Quantification of these data revealed no effect of EE treatment on either parameter (Fig. 3C,D). Expression of Esr1, the gene encoding ERα, was also unaffected by EE treatment (Fig. 3E).

## 2 Experimental design, materials and methods

### 2.1 Animal husbandry and necropsy

Timed pregnant female CD-1 mice (Charles River Laboratories, Raleigh, NC) were housed as described previously [1]. All experimental procedures were approved by the University of Massachusetts Institutional Animal Care and Use Committee.

On pregnancy day 8, dams were randomly assigned to treatment groups. From pregnancy day eight until weaning (on lactational day [LD] 21), pregnant females were weighed daily and fed a small wafer (Nabisco, East Hanover, NJ) treated with ethinyl estradiol (EE) or vehicle alone (70% ethanol) every day. Wafers were dosed with solutions to deliver 0, 0.01 or 1 µg EE/kg/day (Sigma Aldrich, St. Louis, MO; >98% purity; n=12–16 for each dose). EE dose was adjusted for dam body weight daily. Litters were culled to 10 pups on LD1, and pups were weaned on LD21. The dams evaluated in this study were euthanized at LD21. The 0.01 µg/kg/day dose was selected because it has previously been shown to disrupt estrogen-sensitive endpoints in exposed offspring [2]. The higher 1 µg/kg/day dose was selected because it can induce uterotrophic responses in pubertal females and is ~2x higher than the concentrations in prescription birth control pills [3]. Other results from these same females, including effects on behavior and brain, have been submitted for publication elsewhere [4].

On LD21, dams were euthanized via CO_2_ inhalation. From every dam, the right fourth inguinal mammary gland was dissected from the skin, spread on a glass slide (Fisher Scientific, Pittsburgh, PA) and fixed in neutral buffered formalin (10%) (Fisher Scientific) overnight (standard whole mount preparation). The right fifth inguinal mammary gland was fixed in neutral buffered formalin (10%) overnight for histology.

### 2.2 Whole mount mammary gland preparation and analysis

After fixing in neutral buffered formalin, whole-mounted mammary glands were processed through an alcohol series, defatted with toluene, stained with Carmine-alum, dehydrated in an alcohol and xylene series, and preserved in k-pax heat sealed bags (Fisher Scientific) with methyl salicylate (Acros Organics, Morris Plains, NJ) [5]. Digital images of whole-mount mammary glands were obtained using a Zeiss AxioImager dissection microscope (Carl Zeiss Microscopy, Jena, Germany) at 6X magnification and a Zeiss high-resolution color camera. To evaluate the density of epithelial structures in the whole mount, four identical boxes (each 5 mm^2^) were placed to the anterior, posterior, and lateral sides of the lymph node. ZEN software (Carl Zeiss Microscopy) was used to measure the intensity of the mammary tissue in each box; higher numbers indicate less-dense mammary tissue.

### 2.3 Histological analysis of mammary glands

Mammary glands were processed for histological and immunohistochemical evaluation using methods described previously [5]. Digital images were collected using a Zeiss Axio Oberserver.Z1 inverted microscope, a 20X objective, and a high-resolution color camera (Carl Zeiss Microscopy). To quantify the fraction of the mammary gland comprised of adipose tissue versus lobuloalveolar units (lobules), three images were taken at random from non-overlapping areas of the histological section. An identical 10×13 grid was placed on each image, and the tissue type located at each crosshair (lobule, adipose, blood vessel, other connective tissue, etc.) was recorded. To quantify the average lobule size, a minimum of three non-overlapping images were collected using a 40X objective. For each image, an identical 5×5 grid was placed and all lobules that fell on crosshairs were measured for area. A minimum of 9 lobules were evaluated to calculate the average lobule size in each gland.

### 2.4 Immunohistochemistry

Expression of two markers was evaluated using standard methods for immunohistochemistry [5] and commercial antibodies including rabbit anti-ERα (EMD Millipore, Cat# 06–935, Temecula, CA) and rabbit anti-Ki67 (Fisher Scientific, Cat# RM-9106-S1), a marker of proliferation. Sections were incubated with primary antibodies (diluted 1:1000), secondary antibody (goat anti-rabbit, Abcam, Cat# ab64256) and streptavidin peroxidase complex (Abcam, Cat# ab64269). Diaminobenzidene (DAB) chromogen (Abcam, Cat# ab64238) was used to visualize reactions. Sections were counterstained with Harris’ hematoxylin (Fisher Scientific).

Two non-overlapping images were taken of each sample for each marker of interest (ERα, Ki67) with a Zeiss Axio Observer.Z1 inverted microscope. Expression of each marker was evaluated by counting at least 200 epithelial cells. Expression of each marker was expressed as a percent ratio of the total number of epithelial cells evaluated.

### 2.5 qPCR

Total RNA was extracted from mammary glands of individual mice using Trizol reagent (Ambion, Carlsbad, CA) and a BeadBug microtube homogenizer (Sigma Aldrich, St. Louis, MO). Total RNA was quantified by UV spectrophotometry (Nanodrop 1000; Thermo Scientific). One microgram of RNA from each sample was reverse transcribed to cDNA using reverse transcriptase (Applied Biosystems, Inc). The FastStart Universal SYBR Green Master kit (Roche Diagnostics Corporation, Indianapolis, IN) was used for qPCR along with 1 μL of cDNA and 300 nM forward and 300 nM reverse primers for each target gene. β-actin was used as a housekeeping gene. Every sample was run in duplicate for each gene target. The thermal profile was as follows: 10 min at 95 °C; 40 cycles of 15 s at 95 °C, 30 s at 60 °C, and 15 s at 72 °C; a melting-curve analysis was conducted to identify nonspecific products. Relative quantification was determined using the ΔΔCt method to correct for differences in β-actin [6].

### 2.6 Statistical analysis

All analyses were conducted by observers blind to the treatment groups. Data were analyzed using SPSS Version 23 using 1-way ANOVA General Linear Model analyses with treatment as the independent variable, followed by Bonferroni post hoc tests. Data were considered statistically significant at p<0.05. Graphs illustrate means ± standard error.
